# Understanding the Impact of Competitive Advantage and Core Competency on Regional Tourism Revitalization: Empirical Evidence in Taiwan

**DOI:** 10.3389/fpsyg.2022.922211

**Published:** 2022-05-20

**Authors:** Chaohsien Lee, Chihkang Wu, Din Jong

**Affiliations:** ^1^Department of Tourism Management, Business Intelligence School, National Kaohsiung University of Science and Technology, Kaohsiung City, Taiwan; ^2^Business Intelligence School, National Kaohsiung University of Science and Technology, Kaohsiung City, Taiwan; ^3^Department of Digital Design and Information Management, Chung Hwa University of Medical Technology, Tainan City, Taiwan

**Keywords:** regional revitalization, strategic alliance partner selection, competitive advantage, core competency, alliance performance

## Abstract

Competitive advantage and core competency are the unique capabilities and assets of an organization to provide valuable products or services to customers, thus giving the organization a better competitive position in the market than its competitors. In addition, how to create a competitive advantage is also one of the main objectives of business strategy. Therefore, this study focuses on understanding the decisive factors in regional revitalization and the relationship between business strategy, strategic alliance, and alliance performance through small and medium enterprises (SMEs) in Taiwan. This study selected a sample of 220 SMEs in Taiwan that had participated in the SME regional revitalization counseling program. The results showed that competitive advantage, core competency and strategic alliance partner selection had significant effects on alliance performance. In addition, core competency had an indirect effect on alliance performance through strategic alliance partner selection. However, competitive advantage did not have a significant effect on strategic alliance partner selection. Finally, this study proposes management implications and practical suggestions for SMEs’ competitive advantage, core competency, and alliance performance.

## Introduction

In today’s intensely competitive business environments, it is vital for enterprises to adopt swift actions that guarantee their financial security and market position. Many enterprises are constantly employing measures to acquire sustainable competitive advantages. They need to rely more on their internal advantages to provide more customer value and differentiate and expand their products. In other words, an enterprise’s performance is dependent on their core competence. Thus, regional revitalization strategies should shift from offering competitive products or services to focusing on an enterprise’s core competence. Core competence is essential in strategy formulation as it is an important source for acquiring overall competence. Because a wide range of employment opportunities is concomitant with developments in the industrial and commercial sectors, a large proportion of youths and employed individuals live in urban areas, which results in widespread urbanization in many countries. To redress the imbalance between urban and rural development derived from overpopulation in metropolitan areas, Taiwan drew lessons from Japan’s experience of promoting regional revitalization, with the hopes of tapping into unique resources in local areas and creating distinct core values in these areas. In 2016, Taiwan initiated the Regional Revitalization by Design program by incorporating creativity, innovation, and entrepreneurship mechanisms as new drivers for developments in regional industries.

It is important for regional revitalization organizations to leverage their distinct skillsets (core competencies) to distinguish themselves from their competitors. An organization with core competencies is significantly superior to their industry rivals because their core competencies function as a basis for adding value to their products (Wheelen et al., 2018). Core competencies are an organization’s collective learning that involves the means to consolidate various production skills and techniques. Core competencies are a form of communication, participation, and strong organizational commitment. Few companies are able to play the role of a global leader in more than five to six core competencies (Bani-Hani, 2021). Core competencies comprise the strategies for business developments and lead core products and innovative products, which enhances a company’s business performance (Yang, 2015).

The salience of the concept of core competency has prompted researchers to propose various core competence models to help sustain a company’s competitive advantage (Hafeez et al., 2002; Petts, 2015). The study by Srivastava (2005) revealed that core competencies are the foundation for all competitive advantages. Banerjee (2003) contend that core competencies concern the success or failure of knowledge resource recommendation. The definition of core competency is directly associated with performance, according to some researchers such as Chen and Wu (2007), who argue that core competency is a company’s capability to operate efficiently in business environments and deal with various challenges. The differences in the capabilities of companies to select, construct, leverage, and protect their core competencies may shape the differences in their business performance. The concept of core competency is well-developed and is used to assist companies in empowering their identification and utilization of their organizational strengths in a more efficient manner. Previous studies surmised that core competencies are accumulated over time and have a slower rate of change than product and market changes (Gupta et al., 2009; Agha et al., 2012).

Summarizing the arguments above, core competency refers to an organization’s capability to modify, evolve, or combine technologies or knowledge throughout the process of regional revitalization so as to surpass their competitors. Additionally, a company’s core competency is not only their main business strategy but also affects their competitive advantage and business performance. In today’s knowledge-based economy, companies face major changes in competitive environments, such as quantum leaps in technological innovations as well as unpredictable market changes. When companies perceive that they are unable to adapt to the rapid changes in their industry’s environments because they lack the resources and capabilities for independent development, they must consider establishing partnerships by using their connections in order to expand their competitive advantages in fiercely competitive scenarios, acquire necessary organizational resources and assets, and strengthen their competitive advantage and profit margin (Holm et al., 1999; Gulati et al., 2000). Drucker (1995) suggested that knowledge is the most important economic resource for competitive advantage. This collective knowledge lies within the mindset of suppliers, employees, and customers (Mahr et al., 2014), and is also the most important resource that ensures a company’s stable growth. It even surpasses the importance of traditional productivity factors, such as land, capital, and workforce, emphasized by Grossman (2006).

Many companies today embrace agile methodologies to sustain their competitive advantage. Thus, in response to this new competitive challenge, many companies have begun to enhance their core competencies by using different strategic activities or tools to enhance their competitiveness. To a certain degree, competitive advantage is sourced from rare, valuable, and sustainable resources and is shaped by a company’s competitiveness and capabilities (Wernerfelt, 1984; Barney, 1991). Companies should dedicate themselves to developing unique resources. Decision makers generally agree that developing new investments and innovative products can stimulate a company’s growth and shape its competitive advantage (García-Manjón and Romero-Merino, 2012).

Some young companies emphasize technology as their source of competitive advantage, while others opt to use other measures to pursue similar goals. For example, product or service differentiation can be realized through various means, such as technology, design, or workmanship (Gabrielsson et al., 2008). Because every company is distinct and is an expert in its own field (Amit and Schoemaker, 1993; Keupp et al., 2012), not all young entrepreneur companies opt to develop their competitive advantage by building unique skillsets, especially when this strategy is a high-risk and resource-intensive activity (Li and Atuahene-Gima, 2001).

This study suggests that competitive advantage is a company’s ability to leverage its distinct capabilities and resources and provide customers with valuable products or services, thus allowing it to gain a more favorable competitive position than its competitors in the regional revitalization market. The creation of a company’s competitive advantage is also one of the primary objectives of a business strategy as it affects an organization’s business performance. Brondoni (2010) indicated that competition in modern businesses refers to the competition between alliances rather than the competition between companies, as previously perceived. A strategic alliance is an organizational design in which companies cooperate with one another to acquire the resources they need. Thus, companies in the alliance can focus on their expertise and create value by linking the influx of resources between their partners (Varadarajan and Cunningham, 1996; Chan et al., 1997). By cooperating with other companies and sharing existing resources or assets, organizations that lack competitive resources are more eager to partner up with other organizations (Ahuja, 2000). In this regard, a strategic alliance is an important measure that ensures knowledge enhancement and resource usability. Throughout the process of strategy implementation, improper partner selection may increase the risk of opportunism between allies, which restricts cooperation and may even create fractures within the alliance. Thus, the selection of strategic partners is extremely important (Qing et al., 2012).

Following the decision to join a strategic alliance, the next critical decision for a company is to select suitable partners (Hitt et al., 1995). Previous studies have shown that the failure of numerous strategic alliances can be attributed to partner selection. Companies must select suitable partners to acquire value from the strategic alliance (Reuer, 1999). Researchers have identified a wide range of criteria and factors to ensure success in partner selection (Moss Kanter, 1994; Hagen, 2002; Bierly and Gallagher, 2007; Shah and Swaminathan, 2008; Wu et al., 2009). These criteria and factors are verified as key determinants of the performance of a company or strategic alliance (Abuzaid, 2014).

Previous studies have explored the topic of partner selection in a strategic alliance (Geringer and Hebert, 1991; Child et al., 2005), mostly because the process of forming an alliance is considerably attractive to many companies (Geringer and Hebert, 1991; Hitt et al., 2000; Tatoglu, 2000; Wang and Kess, 2006; Doherty, 2009). However, during the pre-alliance partner selection process, many companies assume that there will be many candidate partners to choose from. In reality, the company or organization that initiates the strategic alliance may find it challenging to attract partners to the alliance (Borch and Solesvik, 2016). Researchers in the field of strategy management contend that selecting suitable partners is the key determinant of a strategic alliance’s success (Dong and Glaister, 2006; Shah and Swaminathan, 2008; Wu et al., 2009; Solesvik and Westhead, 2010). A wide body of research has demonstrated that around 60% of strategic alliances fail (Beamish, 1985; Das and Teng, 2003). Thus, the selection of suitable partners is vital for the success of a strategic alliance (Gulati, 1998).

The most important factor in a strategic alliance is the selection of suitable partners, as this enables a holding company to achieve success in dynamic business environments. It is impossible even for high-level alliance management to overcome the preliminary screening of unsuitable partners as well as work selection (Ashayeri et al., 2012). Even though there are many factors that contribute to the successful implementation of a strategic alliance, the importance of partner selection must be emphasized (Medcof, 1997; Ding et al., 2013). Based on existing methods of cooperation and the capabilities of companies and their partners, as well as the use of such capabilities, partner selection should follow systematic guidelines and feasible criteria to minimize the risk of outsourcing. Regarding systematic methods, many studies (Hoffmann and Schlosser, 2001; Sampson, 2004) recommend that companies should verify their motivations before selecting suitable alliance partners. Many theoretical studies on strategic alliances have pointed out that partner selection may affect the business performance of a strategic alliance (Medcof, 1997). Thus, this study included partner selection within the analytical model to examine its effects on the business performance of a strategic alliance.

Horizontal and vertical integrations have blurred the line of industry boundaries. Amidst the backdrop of the globalized division of labor, as well as cooperative environments, strategic alliances have gradually become a mainstream organizational strategy (Das and Teng, 2003). The goal of forming a strategic alliance is to create economic value for partner companies (Dyer and Singh, 1998; Khanna, 1998; Anand and Khanna, 2000; Zollo et al., 2002; Lunnan and Haugland, 2008). Business models have gradually shifted toward strategic alliance formation, with the hopes of achieving synergy, resource intervention, and mutually beneficial alliance performances. Das and Teng (2003) illustrated the attributes of an alliance (collective strength, inter-partner conflicts, and interdependencies) and found that collective strength is a key determinant of alliances’ business performance. The more collective strength an alliance has, the more satisfying their business performance. This implies that companies who solely rely on their own strength are incapable of competing against the collective strength of multiple companies. Thus, the elevation of an alliance’s business performance, as well as the achievement of one another’s objectives, is dependent on the collective strength of partner companies. Alliance cooperation is affected when partners have incompatible goals. Having differing organizational cultures also increases the risk of inter-partner conflict and, thus, affects the alliance’s business performance. Additionally, an alliance’s formation is dependent on mutual support between companies. Partners who are less dependent on the alliance will naturally reduce the value of the alliance’s existence, which leads to its dissolution when it is no longer able to maintain its integrity. Even though the global number of newly-formed alliances is steadily increasing, the failure rate of strategic alliances remains high at 70% (Bleeke and Ernst, 1991; Park and Ungson, 2001; Duysters et al., 2011). This is due to the antecedent of the lack of academic focus on inter-organizational trust (Gulati and Sytch, 2008). Thus, researchers have reviewed previous studies and systematically verified the complex associations between the antecedent variables, so as to explain the differences observed between alliance outcomes (Robson et al., 2006; Ren et al., 2009; Reus and Rottig, 2009; Christoffersen, 2013). Even though these studies, in general, significantly provide generalizable solutions, alliance performance differs greatly based on the features of participating companies (Franco and Haase, 2013; Veiga and Franco, 2015). For example, small and medium enterprises (SMEs) have comparatively different resources and firm sizes than large companies, and their alliance performance is driven by the company’s distinct features (Pansiri, 2008). Some preceding factors only influence the performance of SME alliances, while others may be common regardless of the alliance members’ size. Therefore, the antecedent variables that determine the regional revitalization performance of alliances consisting of SMEs is an important issue.

Based on the aforementioned background and motivations, the decisive factors of an SME’s success in the regional tourism revitalization field are important for understanding the relationships between business strategies, strategic alliances, and alliance performances. For instance, the means to establish a company’s core competency and competitive advantage, as well as partner selection, are important for enhancing the performance of strategic alliances of SMEs in the field of regional tourism revitalization. This study centers on the relationships between core competency, competitive advantage, and strategic alliance partner selection, as well as their influences on alliance performance. The main objective of this study is to understand the extent to which core competency, competitive advantage, and partner selection affect the performance of strategic alliances comprising SMEs participating in regional tourism revitalization, as well as the relationships between these variables.

## Literature Review

### Competitive Advantage

Narver and Slater (1990) suggested that market orientation should be the core focus of a company so as to consistently impart excellent value to customers and gain sustainable competitive advantage. In the meantime, core competency is an enduring capability of an organization (Tampoe, 1994). Barney (1991) and Amit and Schoemaker (1993) agreed that competitive advantage stems from the value, rarity, inimitableness, and irreplaceability of company resources. Tampoe (1994) suggested that core competitive advantage is vital for an organization’s survival. To competitors, it is intangible and inimitable; to a company, it is an exclusive composite of skills, resources, and processes.

McDermott (2003) suggested that core competency confers leading competitive advantage to an organization and creates significant customer value. Regarding indigenous culture tourism, business emeritus should identify their own unique and inimitable core resources and capabilities to create value and benefit both the indigenous tribes and tourists. On the formation of the core competency of the indigenous culture tourism industry, Pearce et al. (2003) noted that core competency is an organization’s sustainable capability or skill acquired by defining, accumulating, and developing their competitive advantage. Talaja et al. (2017) stated that market orientation may strengthen a company’s competitive advantage and subsequently improve their business performance. In the Malaysian manufacturing sector, positive interdependent effects exist between market orientation and competitive advantage even in SMEs (Zhou et al., 2009; Safarnia et al., 2011; Herman et al., 2018). Competitive advantage can therefore be defined as the collection of various items that provide a unique and superior position for companies to distinguish themselves from their competitors in the market (Udriyah et al., 2019).

Additionally, in environmental management practice, companies can improve their competitive advantage through innovation (such as developing green products or processes) or positively affect their competitive performance through the mediating effects of images and media (Chen et al., 2006; Chang, 2011). Additionally, in corporate social responsibility practice, companies can actively influence their competitive advantage by enhancing their relations (Madueno et al., 2016) or customer satisfaction (Saeidi et al., 2015). The fundamental theory of these studies is that a company can obtain competitive advantage by improving their relations with various stakeholders as well as elevating their customer satisfaction.

### Core Competency

The concept of core competency has evolved from a resource-based perspective. It is also known as organizational capability, distinct capability, or dynamic capability. Core competency has been studied extensively (Dierickx and Cool, 1989; Itami and Numagami, 1992; Teece et al., 1997). Companies acquire their competitive advantage by focusing on their core competency and doing what they do best (Srivastava, 2005). Core competency is a multidimensional concept that is often difficult to define, which makes it difficult to distinguish and measure (Hafsi and Thomas, 2005; Ljungquist, 2007; Schreyögg and Geiger, 2007). In business management, the concept of core competency profoundly influences theoretical and practical business strategy management (Duan, 2019).

Gilgeous and Parveen (2001) suggested that core competency is a unique combination of a company’s knowledge, skills, and techniques. Core competency has distinct functions that typically cover various products or markets (Hafeez et al., 2002). Pearce et al. (2003) pointed out that core competency is an organization’s sustainable capability or skill acquired by defining, accumulating, and developing their competitive advantage. Regarding the core competency of companies, Le (2019) suggested that companies are essentially a composite of knowledge and capabilities, and achieving growth in a company’s capabilities is by no means a simple task. Instead, it is a continuous process that is central to the company. The application and scalability of core competency should be achieved through diversified management. The core competency of a company is the essence of diversified strategies. Mooney (2007) defined core competency as an important capability crucial for value creation that involves not only resource ownership. Ljungquist (2008) pointed out that core competency was first conceptualized to validate task diversification in large companies as well as to support internal process tools, such as product development. Gupta et al. (2009) suggested that core competencies are a form of communication, participation, and strong organizational commitment to cross-organizational tasks. Hsu et al. (2014) contend that core competency is a series of distinct and identifiable activities that a company performs in response to technical changes. Core competency is often regarded as the outcome of collective learning and is realized in business activities and processes.

Meanwhile, core competency is also a set of advantages, experiences, knowledge, and capabilities that a company leverages to distinguish itself from its competitors. Employees should instill these qualities to achieve task objectives. Core competency is, therefore, a collection of resources, knowledge, skills, information, and values that not only includes the integration and application of existing knowledge, resources, and skills, but also involves having an acute awareness of market demands, accurate assurance of market opportunities, and providing timely customer satisfaction and valuable services (Duan, 2019).

### Partner Selection of Strategic Alliance

The high level of unpredictability in customer demands and market demands, in addition to increasingly intense global competition as well as environmental uncertainties, has impacted companies tremendously. Therefore, many companies have begun to encourage strategic partnership relations and alliances. On a global scale, business partnerships and alliances are constantly growing (Möller et al., 2005; Gomes et al., 2016). Strategic alliances across companies have become increasingly common. In reality, over the past decades, the number of inter-organizational relationships has grown exponentially. In addition to strategic alliances, researchers have proposed concepts such as the investment portfolio (Wassmer, 2010), strategy networking (Möller and Halinen, 1999; Gulati et al., 2000; Möller and Svahn, 2003; Möller et al., 2005; Möller and Rajala, 2007; Cimon, 2013), relationship maps (Dyer and Singh, 1998), and business networks (Ritter et al., 2004). Nonetheless, strategic alliances and alliance management skills are regarded as important sources of competitive advantage (Dyer and Singh, 1998; Ireland et al., 2002; Schreiner et al., 2009).

Strategic alliances are typically defined as voluntary cooperative arrangements between companies in which they exchange, share, or jointly develop capital, techniques, or company-specific resources (Parkhe, 1993; Gulati, 1998). Traditionally, these arrangements are broadly identified as important measures for creating customer value and enhancing a company’s competitive advantage. These measures include entering new markets and new techniques, developing new products, as well as economies of scale and economies of scope (Mariti and Smiley, 1983; Hagedoorn, 1993). However, it is evident that research and interest in knowledge sharing within strategic alliances and between network members has grown tremendously in recent years (Simonin, 2004). A strategic alliance is defined as a voluntary arrangement in which companies exchange, share, or jointly develop products, techniques, or services (Gulati, 1998). It can also be the cooperative relations that logically drive strategic resource demands and social resource opportunities (Das and Teng, 2000). The goal of strategic alliance forming is to improve effectiveness and competitive status through resource sharing and joint use (Hitt et al., 2000).

Partner selection is an important determinant of an alliance’s success in the process of supply chain management, especially in long-term relations. The study by Huang and Farn (2007) examined information collection behaviors in supply chain partner selection from the perspective of resource dependence and organization inertia. Such behaviors can confer satisfactory cooperation outcomes. The study found a negative correlation between organizational inertia and information collection behaviors, as well as a positive correlation between information collection behaviors and alliance outcomes.

### Alliance Performance

Alliance performance is a subjective measure of an alliance’s cooperation outcomes. It reflects the degree to which preset objectives or planned periodic objectives are achieved, as well as alliance partners’ expectations for the future. Strategic alliances contribute to a company’s competitive advantage by validating its performance outcomes (Baum et al., 2000; Musarra et al., 2016). Participating in different forms of cooperation and partnership in strategic alliances enable startup companies to build trusting relations, enhance their reputation (Jiang et al., 2015), and expand their market presence (Park et al., 2002). The better the alliance outcomes, the more benefits a company acquires, and the more willing it is to continue its cooperation with alliance partners. In other words, companies who highly value their cooperation with alliance partners are more willing to sustain their cooperative relations. In contrast, when undesirable alliance outcomes arise, partners may doubt the current status of their cooperative relations, calculating the differences between the outcomes and their expectations.

According to Bouncken et al. (2015), performance indicators and alliance objectives are important and strongly correlated. The performance of a strategic alliance is ultimately measured based on the goals of alliance formation (Franco, 2011; Christoffersen et al., 2014). In reality, the assessment criteria of alliance performance differ from those of company performance. This has been empirically supported in research, which amplifies the need for objective measures, including relationship-oriented measures and performance-related measures (such as measures with financial qualities) (Ren et al., 2009; Franco, 2011). Pirela (2007), Franco and Haase (2013), and Ahn et al. (2015) agreed that the shared resources within a strategic alliance enhance the entrepreneurial directions of alliance members. In other words, innovation competence, risk acceptance, and objectivity affect organizational performance. Albers et al. (2013), Li et al. (2013), and Skålholt and Thune (2014) suggested that a strategic alliance enables companies to access the technologies that they do not possess, which is vital for enhancing their business performance and sustaining their competitive advantage.

## Research Methodology

### Hypotheses Development

A study by Solesvik and Westhead (2010) examined the company reports of Norwegian maritime companies to analyze their partner selection criteria for forming strategic alliances and how these criteria enhance their competitive advantage. A study by Qing et al. (2012) validated the relationships between partner selection criteria and innovation performance of technological standard alliances in China. Tsou et al. (2015) mentioned that the four criteria of business partner selection affect the joint innovation of the provided services and, subsequently, the competitive advantage of a company. Therefore, selecting suitable partners is vital for the success of a strategic alliance aimed at developing green innovations as this ensures the company’s competitive advantage. Based on the arguments above, this study proposes Hypothesis 1 as follows.

**H1: Competitive advantage has a significant and positive effect on strategic alliance partner selection.**

Bronder and Pritzl (1992) pointed out that a company can achieve or acquire systematic capabilities in certain markets through strategic alliances. Morrison and Mezentseff (1997) argued that strategic alliances should be able to support and leverage core competencies. The strength of the core competencies of candidate partners is among the most important determinants of their success as a supply chain member. Based on the arguments above, this study proposes Hypothesis 2 as follows.

**H2: Core competency has a significant and positive effect on strategic alliance partner selection.**

Following its increasing popularity and prominence in recent years, the influence of alliance networks on the overall performance of a company has become a popular topic for researchers and businesses (Gulati et al., 2000; Koka and Prescott, 2002). Suitable alliance partners and motivations confer better alliance outcomes (Cravens et al., 2000). Thus, the characteristics of partners indeed affect alliance performance. Inter-partner coordination is a key indicator of alliance performance. Furthermore, previous studies have indicated that alliance performance is directly determined by partner selection (Mohr and Spekman, 1994).

The study by Solesvik and Westhead (2010) revealed that vigilant partner selection is a factor that determines a company’s success in a strategic alliance. Other studies also note that successful strategic alliances are associated with building trusting and honest relationships, having partners with common strategic goals, and having partners who provide necessary resources and capabilities. In a study on the partnerships between SMEs, Liu (2021) found that partnership quality positively affects company performance. Based on the arguments above, this study proposes Hypothesis 3 as follows.

**H3: Strategic alliance partner selection has a significant and positive effect on alliance performance.**

Udriyah et al. (2019) analyzed 150 textile SMEs in Malaysia and found that market orientation and innovation positively and significantly affect competitive advantage. Market orientation and innovation contribute to competitive advantage, and competitive advantage partially confers positive and important effects on business performance. Market orientation and innovation also, directly or indirectly, confer major effects on business performance through competitive advantage. Other researchers (Agha et al., 2012; Cantele and Zardini, 2018; Udriyah et al., 2019; Falahat et al., 2020; Maziriri, 2020) have demonstrated the positive effects of competitive advantage on organizational performance. Thus, Hypothesis 4 is proposed as follows.

**H4: Competitive advantage has a significant and positive effect on alliance performance.**

Previous studies have identified a relationship between competitive advantage and organizational performance (Agha et al., 2012). Core competencies are measured through shared vision, cooperation, and empowerment. The study by Agha et al. (2012) also noted that even though core competency strongly and positively affects competitive advantage and organizational performance, competitive advantage also significantly affects organizational performance. The study demonstrated the salience of core competency on competitive advantage and organizational performance. To maintain their competitiveness and gain competitive advantage, company managers can attempt to enhance their organizational performance, cooperation, and empowerment by managing the various components of core competency (i.e., shared vision). In a study on airport shopping centers, Liang et al. (2013) revealed that core competency is positively correlated with organizational performance. Since core competency positively affects organizational performance, this study proposes Hypothesis 5 as follows.

**H5: Core competency has a significant and positive effect on alliance performance.**

### Measurement Items

Core competency consists of shared vision, cooperation, and empowerment. The 23-item core competency scale in this study was adapted from the scale developed by King and Zeithaml (2001). The 11-item competitive advantage scale was adapted from the scales developed by Macmillan and Tampo (2000), and covers sensitivity and responsiveness. The 14-item partner selection scale was adapted from the scale developed by Abuzaid (2014) focusing on alliance partner characteristics, which covers compatibility, complementarity, and commitment. The six-item alliance performance scale was developed according to the studies by Mohr and Spekman (1994), and covers satisfaction with the alliance, attainment of objectives, and satisfaction with achievements. All items were measured on a seven-point Likert scale ranging from “strongly disagree” (1 point) to “strongly agree” (7 points).

### Sampling and Subjects

Booming industrial and commercial developments in Taiwan have created numerous job opportunities. A majority of young employed workers live in urban areas, which amplifies urbanization in Taiwan. This study examines the relationships between the core competency, competitive advantage, and alliance performance of SMEs participating in regional tourism revitalization. The sample size consists of 233 companies that have participated in the Small Business for Township Revitalization program. Two hundred and forty-five questionnaires were administered, eight of which were removed for having incomplete or inappropriate responses. There were 220 valid responses in total, indicating an effective recovery rate of 96.06%. Table 1 presents the means, standard deviations, skewness and kurtosis of measurement items. The skewness (<2) and kurtosis (<7) statistics of each variable reveals that the empirical data did not significantly deviate from normal distribution (Curran et al., 1996).

**TABLE 1:** Descriptive statistics and factor loadings.

Item	Mean	Standard deviation	Skewness	Kurtosis	Factor loading	*T*-value
CA1	6.10	0.90	–0.87	0.27	0.71	18.11
CA2	5.84	1.03	–0.57	–0.69	0.80	28.61
CA3	5.89	0.90	–0.64	–0.02	0.90	59.46
CA4	5.82	0.96	–0.61	–0.33	0.79	19.99
CA5	5.94	0.86	–0.61	0.12	0.85	28.62
CA6	5.68	0.97	–0.44	–0.61	0.79	24.57
CA7	5.83	1.03	–0.55	–0.71	0.87	51.55
CA8	5.82	0.96	–0.54	–0.43	0.76	19.32
CA9	5.83	0.89	–0.55	–0.08	0.77	19.68
CA10	5.71	1.03	–0.39	–0.85	0.86	41.17
CA11	5.73	1.08	–0.4	–1.01	0.82	32.75
CC1	6.42	0.77	–1.24	1.26	0.80	38.83
CC2	6.36	0.87	–1.29	1.01	0.86	44.57
CC3	6.18	0.82	–0.82	0.45	0.82	29.62
CC4	6.05	0.86	–0.74	0.28	0.80	25.23
CC5	6.10	0.89	–0.82	0.17	0.84	34.05
CC6	6.01	0.84	–0.71	0.43	0.73	19.25
CC7	6.15	0.79	–0.67	0.28	0.65	17.33
CC8	5.85	0.91	–0.53	–0.24	0.63	12.32
CC9	5.82	0.83	–0.54	0.29	0.67	14.21
CC10	6.00	0.91	–0.69	–0.07	0.76	25.14
CC11	6.01	0.97	–0.73	–0.28	0.75	24.39
CC12	6.13	0.86	–0.82	0.31	0.81	35.32
CC13	6.17	0.73	–0.48	0.10	0.83	39.39
CC14	5.66	1.05	–0.35	–0.98	0.63	13.32
CC15	5.80	0.88	–0.6	0.05	0.74	21.62
CC16	5.87	0.97	–0.63	–0.35	0.61	13.18
CC17	5.59	1.10	–0.28	–1.17	0.75	21.20
CC18	5.82	0.90	–0.59	–0.06	0.80	32.40
CC19	5.75	0.96	–0.53	–0.45	0.74	23.76
CC20	5.86	1.06	–0.59	–0.77	0.66	16.10
CC21	5.99	0.89	–0.73	0.13	0.76	27.25
CC22	5.80	1.10	–0.57	–0.77	0.76	24.72
CC23	5.87	1.05	–0.63	–0.67	0.84	47.70
PSA1	5.97	0.99	–0.72	–0.34	0.86	47.56
PSA2	6.12	0.97	–0.9	–0.08	0.88	52.99
PSA3	5.64	1.20	–0.79	0.60	0.73	17.39
PSA4	6.13	0.91	–0.88	0.15	0.76	28.54
PSA5	5.80	1.28	–1.86	5.67	0.70	25.32
PSA6	5.59	1.10	–0.24	–1.20	0.72	20.17
PSA7	5.84	1.08	–0.55	–0.89	0.83	43.38
PSA8	5.68	1.10	–0.38	–1.09	0.85	37.99
PSA9	6.19	0.77	–0.64	0.20	0.76	29.16
PSA10	6.03	0.89	–0.73	0.08	0.77	26.90
PSA11	5.94	0.97	–0.71	–0.27	0.78	22.89
PSA12	5.59	1.14	–0.26	–1.30	0.92	72.03
PSA13	5.67	1.14	–0.33	–1.25	0.87	39.44
PSA14	5.96	1.05	–1.05	0.90	0.79	26.55
AP1	5.43	1.05	–0.14	–1.16	0.61	14.04
AP2	5.39	1.06	–0.25	–0.92	0.68	15.43
AP3	5.34	1.10	0.04	–1.30	0.69	15.41
AP4	5.45	1.07	–0.13	–1.19	0.69	16.93
AP5	5.33	1.10	–0.09	–1.24	0.75	21.11
AP6	5.30	1.10	–0.18	–0.72	0.72	17.76
AP7	5.30	1.13	0.07	–1.41	0.71	18.37
AP8	5.31	1.09	–0.01	–1.17	0.66	15.98
AP9	5.30	1.01	–0.18	–1.02	0.74	24.07
AP10	5.55	0.92	–0.37	–0.54	0.75	29.25
AP11	5.44	0.99	–0.21	–0.95	0.79	32.06
AP12	5.38	1.11	0.01	–1.32	0.80	36.99
AP13	5.32	1.03	–0.03	–1.18	0.77	27.25
AP14	5.33	1.01	–0.08	–1.14	0.77	21.89

*CA, competitive advantage; CC, core competency; PSA, partner selection of alliance; AP, alliance performance.*

## Empirical Data Analysis

### Outer Model

**FIGURE 1:**
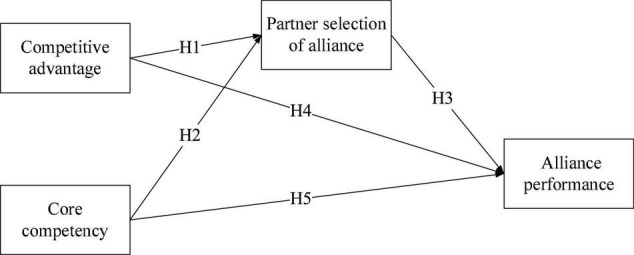
Research model.

To test the proposed research model (see Figure 1) and analyze the formulated hypotheses, Partial Least Square Structural Equation Modeling (PLS-SEM) was used. In this research, SmartPLS 3.3.8 was applied for analyzing data. Previous studies have recommended that outer model can be performed prior to the correlational analysis of a causal effects model, and PLS-SEM analysis can only be performed when the outer model results can accurately reflect the constructs. In terms of convergent validity, Hair et al. (2010) proposed that individual item reliability, composite reliability, and average variance extracted (AVE) must be considered, as good convergence is attained when all three indicators fall within a standard range of values. Chin (1998) suggested that item reliability is acceptable when the standardized factor loading exceeds 0.6 (as shown in Table 1), and is even better when it exceeds 0.6. Hooper et al. (2008) stated that items with a standardized factor loading below 0.45 should be removed because they contain many errors. A questionnaire’s overall reliability is a measure of both its reliability and consistency. Composite reliability is adopted as an indicator of the internal consistency of each variable in this study (as shown in Table 2).

**TABLE 2:** Reliability and convergent validity.

Construct	Cronbach’s alpha	Composite reliability	Average variance extracted
Competitive advantage	0.88	0.84	0.72
Core competency	0.93	0.88	0.72
Partner selection of alliance	0.93	0.916	0.785
Alliance performance	0.93	0.94	0.53

This study adopted the approach proposed by Henseler et al. (2015) to perform discriminant validity testing *via* heterotrait-monotrait ratio of correlations (HTMT). All HTMT ratio values between the constructs are below the conservative threshold of 0.85 (Henseler et al., 2015; Kline, 2015), as shown in Table 3. Therefore, this study has acceptable discriminant validity.

**TABLE 3:** Heterotrait-monotrait ratio of correlations.

Construct	CA	CC	PSA	AP
Competitive advantage				
Core competency	0.835			
Partner selection of alliance	0.615	0.741		
Alliance performance	0.679	0.701	0.696	

*CA, competitive advantage; CC, core competency; PSA, partner selection of alliance; AP, alliance performance.*

### Inner Model

Inner model specifically elucidates the relationships between constructs and can be used to validate the hypotheses in the study framework. First, the path coefficient of the effect of competitive advantage on strategic alliance partner selection is 0.050 (*T*-value = 0.581, *p*-value = 0.561). Thus, H1 is not supported. Second, the path coefficient of the effect of core competency on partner selection is 0.658, with a standard deviation of 0.078 (*T*-value = 8.388, *p*-value = 0.000 < 0.05). H2 is, therefore, supported by the significant level testing. Third, the path coefficient of the effect of partner selection on alliance performance is 0.376 (*T*-value = 5.960, *p*-value < 0.001). H3 is therefore supported. Fourth, the path coefficient of the effect of competitive advantage on alliance performance is 0.249 (*T*-value = 3.266, *p*-value < 0.01). H4 is, therefore, supported. Fifth, the path coefficient of the effect of core competency on alliance performance is 0.226 (*T*-value = 2.582, *p*-value < 0.05). Therefore, H5 is supported.

**FIGURE 2:**
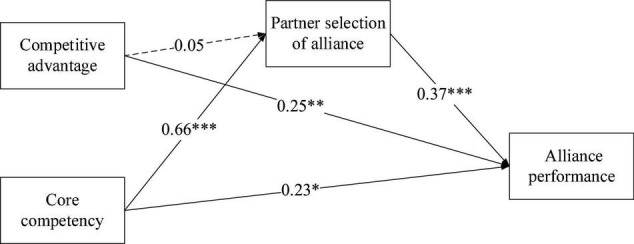
Inner model. *p-value < 0.05; ^**^p-value < 0.01; ^***^p-value < 0.001.

Furthermore, this study used the *R*^2^ statistic as an indicator of the explanatory power of a model (Chin, 1998). Endogenous latent variables with a *R*^2^> 0.67, *R*^2^> 0.33, and *R*^2^> 0.19 indicates high, moderate, and low explanatory power, respectively (Chin, 1998). The results showed that the *R*^2^ of partner selection and alliance performance is 0.486 and 0.562, respectively. This suggests that the model has a decent explanatory power, as shown in Figure 2 and Table 4.

**TABLE 4:** Hypotheses testing results.

Hypothesis	Path	Path coefficient	Standard deviation	T-value
H1	Competitive advantage- > Partner selection of alliance	0.050	0.086	0.581
H2	Core competency- > Partner selection of alliance	0.658***	0.078	8.388
H3	Partner selection of alliance- > Alliance performance	0.376***	0.063	5.960
H4	Competitive advantage- > Alliance performance	0.249**	0.076	3.266
H5	Core competency- > Alliance performance	0.226*	0.088	2.582

*p-value < 0.05; **p-value < 0.01; ***p-value < 0.001.

## Conclusion

This section concludes the statistical analysis findings in the previous section and proposes several recommendations. Firstly, we shall summarize the findings of this study. Next, we will outline the management implications and practical recommendations for SMEs participating in township tourism revitalization to enhance their core competency, competitive advantage, partner selection, and alliance performance. Lastly, we shall describe the limitations of this study and offer several recommendations for future research.

First, the path analysis results showed that competitive advantage did not have a significant effect on partner selection. This implies that there is no significant correlation between competitive advantage and partner selection among SMEs participating in township revitalization. The hypothesis is not supported. A possible reason is that competitive advantage refers to a company’s existing strategies that cannot be replicated by their potential competitors. Companies with strong competitive advantage are less likely to select companies with weak competitive advantage as their partners. On the other hand, a company with weak competitive advantage is less likely to be selected as a partner by other companies with strong competitive advantage. As a result, there is no significant correlation between competitive advantage and partner selection.

Next, the path analysis results showed that core competency has a significant effect on partner selection. This indicates that there is a significant correlation between core competency and partner selection among SMEs participating in regional revitalization. The hypothesis is supported. Core competencies in this study include three internal sub-dimensions of a company, i.e., shared vision, cooperation, and empowerment. During partner selection, it is necessary for companies to consider their shared visions, methods of cooperation, degrees of empowerment, as well as similarities and compatibility with alliance partners. This results in a significant correlation between core competency and partner selection among SMEs participating in township revitalization. This finding validates a previous study, which found that core competency is associated with strategic alliance formation (Morrison and Mezentseff, 1997).

Third, the path analysis results showed that partner selection has a significant effect on alliance performance. This indicates that there is a significant correlation between partner selection and alliance performance among SMEs participating in township revitalization. The hypothesis is supported. Selecting the correct partners enables companies to enjoy synergistic effects that augment their alliance performance. In contrast, selecting unsuitable partners not only encumbers alliance performance but may also lead to personal loss. The success of a strategic alliance hinges on the selection of suitable partners. Therefore, there is a significant correlation between partner selection and alliance performance among SMEs participating in township revitalization, and this finding is in line with a previous study, which found that core competency is associated with strategic alliance and competitive advantage formation (Solesvik and Westhead, 2010).

Fourth, the path analysis results showed that competitive advantage has a significant effect on alliance performance. This indicates that there is a significant correlation between competitive advantage and alliance performance among SMEs participating in township revitalization. The hypothesis is supported. Companies with strong competitive advantage have excellent sensitivity and responsiveness, and are capable of improving their performance. In a similar vein, allying with companies with stronger competitive advantage naturally generates a better alliance performance. Thus, there is a significant correlation between competitive advantage and alliance performance among SMEs participating in township revitalization. This finding is consistent with previous studies, which found that core competency is associated with alliance performance (Cantele and Zardini, 2018; Udriyah et al., 2019; Falahat et al., 2020; Maziriri, 2020).

Fifth, the path analysis results showed that core competency has a significant effect on alliance performance. This indicates that there is a significant correlation between core competency and alliance performance among SMEs participating in township revitalization. The hypothesis is supported. Core competencies in this study include three sub-dimensions intrinsic to a company, i.e., shared vision, cooperation, and empowerment. Company alliances with stronger core competencies will definitely perform better than those with weaker core competencies. Therefore, there is a significant correlation between core competency and alliance performance among SMEs participating in township revitalization. This finding is consistent with a previous study, which found that core competency is associated with alliance performance (Liang et al., 2013).

Although this study has both theoretical and practical implications, there are also limitations that need to be improved in subsequent studies. First, the results of this study may be limited in inference because the level of understanding of the survey respondents in the government-subsidized enterprises may be different from the actual level of understanding of the contents of the completed questionnaire. In addition, the respondents of this study may be subject to differences in industry and employment environment. Therefore, it is suggested that future studies may conduct in-depth discussions on the causes and consequences of industrial alliances through different industrial categories. In addition, future studies may conduct in-depth focus group interviews to better understand the possible factors affecting the performance of business alliances.

## Data Availability Statement

The raw data supporting the conclusions of this article will be made available by the authors, without undue reservation.

## Author Contributions

CL and CW: conceptualization and methodology. CL: investigation and formal analysis. CL, CW, and DJ: writing—original draft preparation and writing—review and editing. DJ: supervision. All authors: contributed to the article and approved the submitted version.

## Conflict of Interest

The authors declare that the research was conducted in the absence of any commercial or financial relationships that could be construed as a potential conflict of interest.

## Publisher’s Note

All claims expressed in this article are solely those of the authors and do not necessarily represent those of their affiliated organizations, or those of the publisher, the editors and the reviewers. Any product that may be evaluated in this article, or claim that may be made by its manufacturer, is not guaranteed or endorsed by the publisher.
